# The implementation of a home-based isometric wall squat intervention using ratings of perceived exertion to select and control exercise intensity: a pilot study in normotensive and pre-hypertensive adults

**DOI:** 10.1007/s00421-023-05269-2

**Published:** 2023-07-17

**Authors:** John W. D. Lea, Jamie M. O’Driscoll, Jonathan D. Wiles

**Affiliations:** School of Human and Life Sciences, Canterbury Christ Church University, Canterbury, Kent UK

**Keywords:** RPE, Perceived effort, Hypertension, Blood pressure, Resistance exercise, Randomised control trial

## Abstract

**Purpose:**

Isometric exercise (IE) and isometric wall squat (IWS) training have been shown to be effective methods of reducing arterial blood pressure. However, most IE interventions require methodologies and equipment that could present a barrier to participation. Therefore, this study aimed to examine the effectiveness of an accessible RPE prescribed IWS intervention.

**Methods:**

Thirty normotensive and pre-hypertensive adults were randomly assigned to a control group or one of two 4-week home-based IWS intervention groups: the first group conducted IWS exercise where intensity was prescribed and monitored using RPE (RPE-EX), whilst the other used a previously validated HR prescription method (HR-EX). Resting and ambulatory heart rate (HR) and blood pressure (BP) were measured pre- and post-intervention.

**Results:**

Minimum clinically important differences (MCID; − 5 mmHg) in SBP and/or DBP were shown in 100% of intervention participants. Statistically significant reductions were shown in resting seated BP (RPE-EX: SBP: − 9 ± 6, DBP: − 6 ± 4, MAP: − 6 ± 3 mmHg; HR-EX: SBP: − 14 ± 6, DBP: − 6 ± 4, MAP: − 8 ± 4 mmHg), supine BP (RPE-EX: SBP: − 8 (− 5), DBP: − 8 (− 7), MAP: − 8 (− 4) mmHg; HR-EX: SBP: − 5 (− 4), MAP − 5 (− 4) mmHg), and ambulatory SBP (RPE-EX: − 8 ± 6 mmHg; HR-EX: − 10 ± 4 mmHg) following the interventions. There were no statistically significant differences between intervention groups in the magnitude of BP reduction.

**Conclusion:**

RPE prescribed IWS exercise can provide an effective and more accessible method for reducing BP at home, providing reductions comparable to the current HR-based prescription method.

**Supplementary Information:**

The online version contains supplementary material available at 10.1007/s00421-023-05269-2.

## Introduction

Hypertension, characterised by a sustained elevation in arterial blood pressure (≥ 140 mmHg systolic and/or ≥ 90 mmHg diastolic), is the most common long-term health condition in the UK and a primary risk factor for mortality (Mancia et al. 2013). It is estimated that one in three people (31% male and 26% female) in the UK have high BP, with approximately 75,000 deaths being attributable to the condition annually (NICE 2019). The importance of lifestyle changes (smoking cessation/alcohol use/diet/exercise/weight loss) for patients with hypertension in the absence of other risk factors should not be overlooked (NICE 2019). Indeed, reductions in BP equal to or greater than the minimum clinically important difference (MCID), − 5 mmHg, are associated with significant reductions in the risk of developing hypertension and cardiovascular disease (Whelton et al. 2002), as well as reduced risk of myocardial infarction, stroke and mortality (NICE 2011).

Exercise has been recommended as a non-pharmacological lifestyle modification for the treatment of hypertension (Brook et al. 2015). Isometric exercise (IE) training has been shown to be an effective methodology to reduce resting (Wiles et al. 2010, 2017) and ambulatory BP (Taylor et al. 2019) equally in both normotensive males and females (Badrov et al. 2016), as well as those with suboptimal BP (Taylor et al. 2019). Moreover, IE results in a greater magnitude of BP reduction when compared to either aerobic or dynamic resistance exercise training (Cornelissen and Smart 2013) for a significantly lower weekly exercise time. To contextualise efficacy, the magnitude of BP reductions following IE training is similar (Naci et al. 2019) or greater (Taylor et al. 2019) than those achieved using antihypertensive medication in hypertensive adults with no contraindicated co-morbidities.

To date, most IE interventions designed to lower BP utilise either handgrip (Stiller-Moldovan et al. 2012), leg extension (Wiles et al. 2010), or wall squat exercise (Taylor et al. 2019). The current methods used to prescribe and control IE intensity require specialist, often expensive (e.g. isokinetic and handgrip dynamometers) equipment, and involve maximal testing by a trained health care professional in a dedicated setting to quantify relative training loads; all of which add barriers to general participation (Lea et al. 2021a). Therefore, if a means of prescribing and monitoring IE intensity could be developed that did not require expensive equipment and specialist testing, these potential barriers could be removed, allowing the benefits of IE training to be utilised by a wider audience for blood pressure control.

Ratings of perceived exertion (RPE) could provide an easy to use and accessible alternative means of assessing and monitoring exercise intensity (Lea et al. 2021a, b, 2022). Indeed, it has long been established that RPE provides an accurate estimation of exercise intensity and physiological exertion during cardiovascular exercise (Chen et al. 2002). In addition, there is now a growing body of evidence that indicates that various RPE scales provide a valid measure of exercise intensity during resistance exercise (Lea et al. 2022) independent of participant age (Pincivero et al. 2010) or sex (Buckley and Borg 2011; Gearhart et al. 2011). Recently, RPE has been shown to provide valid and reliable measurements of exercise intensity and physiological measures of exertion (e.g. HR) during continuous incremental isometric wall squat (IWS) exercise (Lea et al. 2021a); and can discern between workloads (knee joint angles) at the resolution previously shown to be necessary (Wiles et al. 2010, 2017) for IWS prescription to lower BP over a 4-week training period (Lea et al. 2021b). However, no study to date has assessed the effectiveness of an RPE prescribed IE intervention for the reduction of arterial BP.

Therefore, the aims of this research were to: (1) examine the effectiveness of a 4-week home-based IWS training intervention, using RPE to prescribe and monitor exercise intensity, as a means of reducing resting and ambulatory arterial BP; and (2) compare the RPE prescribed intervention with the previously validated and implemented HRpeak method of prescription (Wiles et al. 2017; Taylor et al. 2019).

## Methods

### Participants

Thirty volunteers, 24 pre-hypertensive males and 6 females (4 normotensive and 2 pre-hypertensive), completed this study (age: 18–65 years; stature: 161–197 cm; body mass: 53–109 kg; body mass index [BMI]: 18–34). All participants were former smokers (≥ 6-months) or had never smoked, had Systolic BP (SBP) between 90 and 139 mmHg and diastolic BP (DBP) between 60 and 89 mmHg, and had no injury or illness including no clinical diagnosis of any cardiovascular condition or dysfunction. Five of the female participants were taking the contraceptive pill, no other medications were being taken. Before testing, written informed consent was obtained from all participants and a health and medical questionnaire was completed.

### Study design

**Fig. 1:**
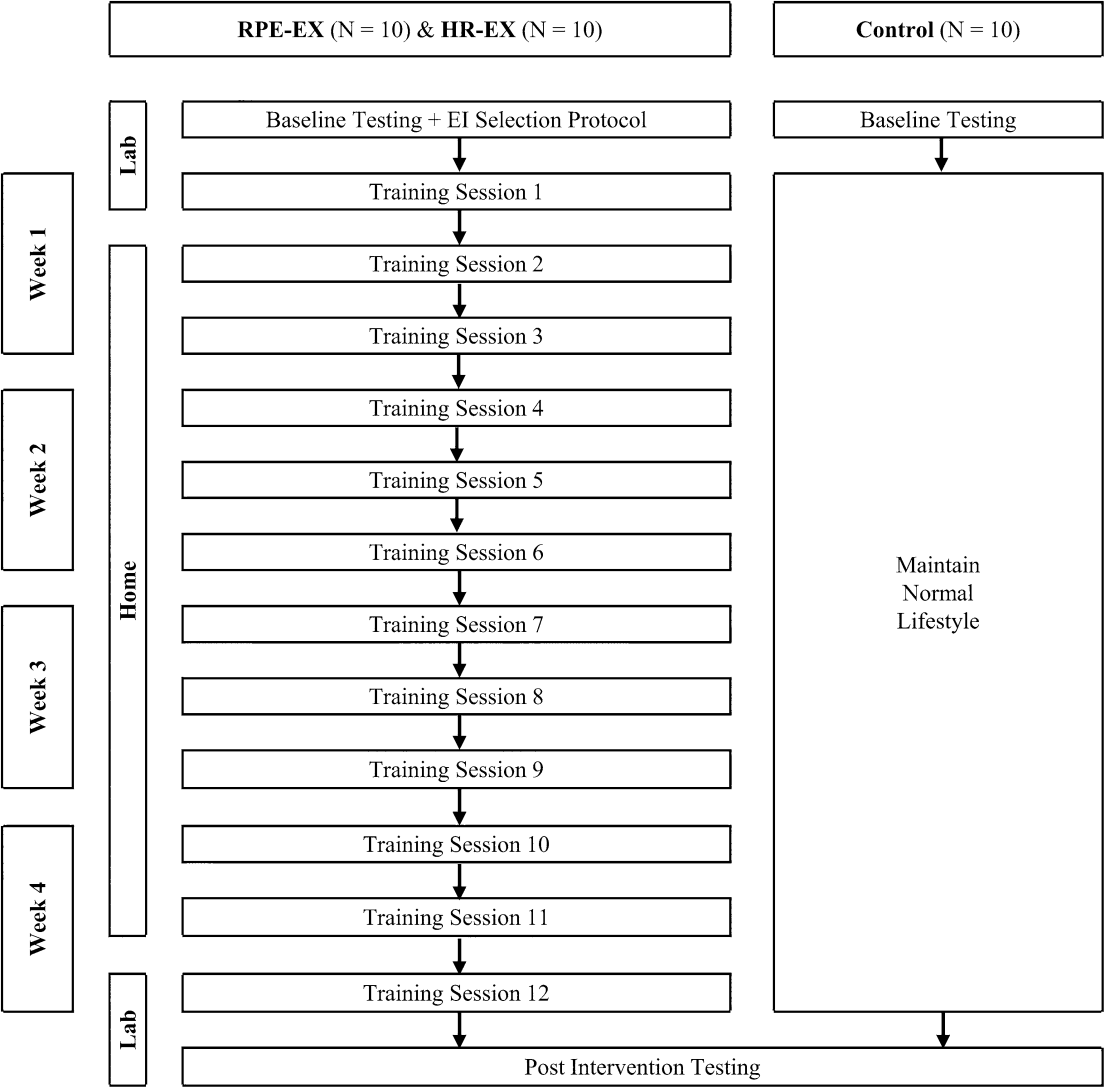
Schematic of the study design and group requirements

This was a randomised control study with within subjects repeated measures and between group comparisons. Before attending the laboratory on the first occasion, participants were randomised in to one of 3 groups (two intervention and 1 control) as shown in Fig. 1. Males and females were randomised separately, to ensure a more even sex split between the 3 groups. Participants allocated to the control group (CON) received no intervention and agreed to attend the laboratory twice, separated by a 4-week period. The intervention groups agreed to attend the laboratory on 4 separate occasions and were required to undertake a 4-week home-based IWS intervention, with exercise intensity prescribed using the 95% HRpeak method (HR-EX), as in Wiles et al. (2017) and Taylor et al. (2019), or with exercise intensity self-prescribed using an RPE selection protocol (RPE-EX). All participants were asked, as far as possible, to continue with their usual diet and exercise routines, in addition to the prescribed isometric exercise. At the start of each laboratory session, participants gave verbal confirmation that they had abstained from food for 4-h, caffeine for 12-h, alcohol for 24-h and strenuous exercise for 24-h. If they had not followed these instructions, the session was re-scheduled. This study was approved by Canterbury Christ Church University’s Ethics Committee (15/SAS/223) and conducted according to the Declaration of Helsinki.

### Procedures

#### Familiarisation

Prior to their first attendance to the laboratory, participants received an information pack outlining the study design, testing protocols, measurement procedures and participants instructions for each of the possible study groups. At the start of the first laboratory session, participants were informed of the group to which they were randomly allocated, and the relevant study protocols, measurements, and requirements were explained to them verbally. As part of this explanation, participants were shown the equipment that would be used and, if relevant, were given a demonstration of the correct wall squat technique. Participants were encouraged to ask questions throughout the familiarisation to ensure that all testing procedures were fully understood before providing informed consent.

#### Resting measures

At the start of each laboratory testing session, participants rested in a seated position for 10 min. After 10 min rest, HR, SBP, DBP and mean arterial pressure (MAP) were recorded using an oscillometric BP monitor on the participants left arm (Dinamap^®^ Pro, GEMedical Systems, Slough, UK). Three measurements were taken, each separated by 1-min (Whelton et al. 2018). If differences between the consecutive measurements exceeded 5 mmHg, then an additional measurement was taken. The mean result for each variable was calculated for analysis and for classification of each participant’s resting HR and BP status in the descriptive data. Normotension was classified as an SBP between 90 and 119 mmHg and a DBP between 60 and 79 mmHg (WHO 2007; Mancia et al. 2013; Public Health England 2014), whilst pre-hypertension was defined as an SBP of 120–139 mmHg and a DBP of 80–89 mmHg (Pickering et al. 2005). Following the seated measurements, participants rested in a supine position for 15 min. After an initial 10-min period, HR and BP were measured continuously for the remaining 5 min using a plethysmographic device (Task Force^®^ Monitor, CNSystems, Graz, Austria). Resting HR and BP values were calculated as the mean of the 5-min supine measurement period. Resting measurements were collected in a quiet room with the lights dimmed (Pickering et al. 2005). Participants were instructed not to talk and to remain as still as possible throughout this period.

#### Ambulatory measures

Following the baseline and post-intervention testing sessions, participants were fitted with an ambulatory blood pressure (AMBP) monitor (Welch Allyn 6100, Welch Allyn Inc, NY, USA). CON participants had the AMBP monitor fitted at the end of the laboratory testing session, whilst the intervention participants were given 24-h to recover from the IWS exercise before the measurements were taken. As such, intervention participants were shown how to fit, turn on, and start the ambulatory BP cuff and were instructed to do so 24–48 h after completion of the preceding laboratory sessions. Once the device was fitted to the participant, it was turned on and a manual measurement was collected. This first measurement initiated a 24-h data collection period. Participants were asked to avoid tight fitting clothing and exercise, and to maintain their normal dietary habits during this 24-h monitoring period. The device was programmed to take measurements every 20–30 min during the day and every 30–60 min at night. The same AMBP monitor was used by each participant for both 24 h AMBP measurements (pre- and post-intervention).

#### Ratings of perceived exertion

RPE was measured in both intervention groups using the Isometric Exercise Scale. Before use, participants received standardised scaling and anchoring instructions, as described in Lea et al. (2021a, b).

### Control group

Participants in the CON group attended the laboratory twice, once at the start of the study and once at the end, to undertake resting and ambulatory cardiovascular measurements, including HR and BP. Following the first testing session, participants were sent home wearing an ambulatory BP cuff for 24 h. After that 24-h period, the participants were required to continue with their normal life and routines for 4-weeks (28 days). At the end of the 4-week period, participants were once again required to attend the laboratory, within a 72-h window, for final resting and ambulatory cardiovascular measurement.

### RPE-EX group

#### Baseline testing and training intensity prescription

**Fig. 2:**
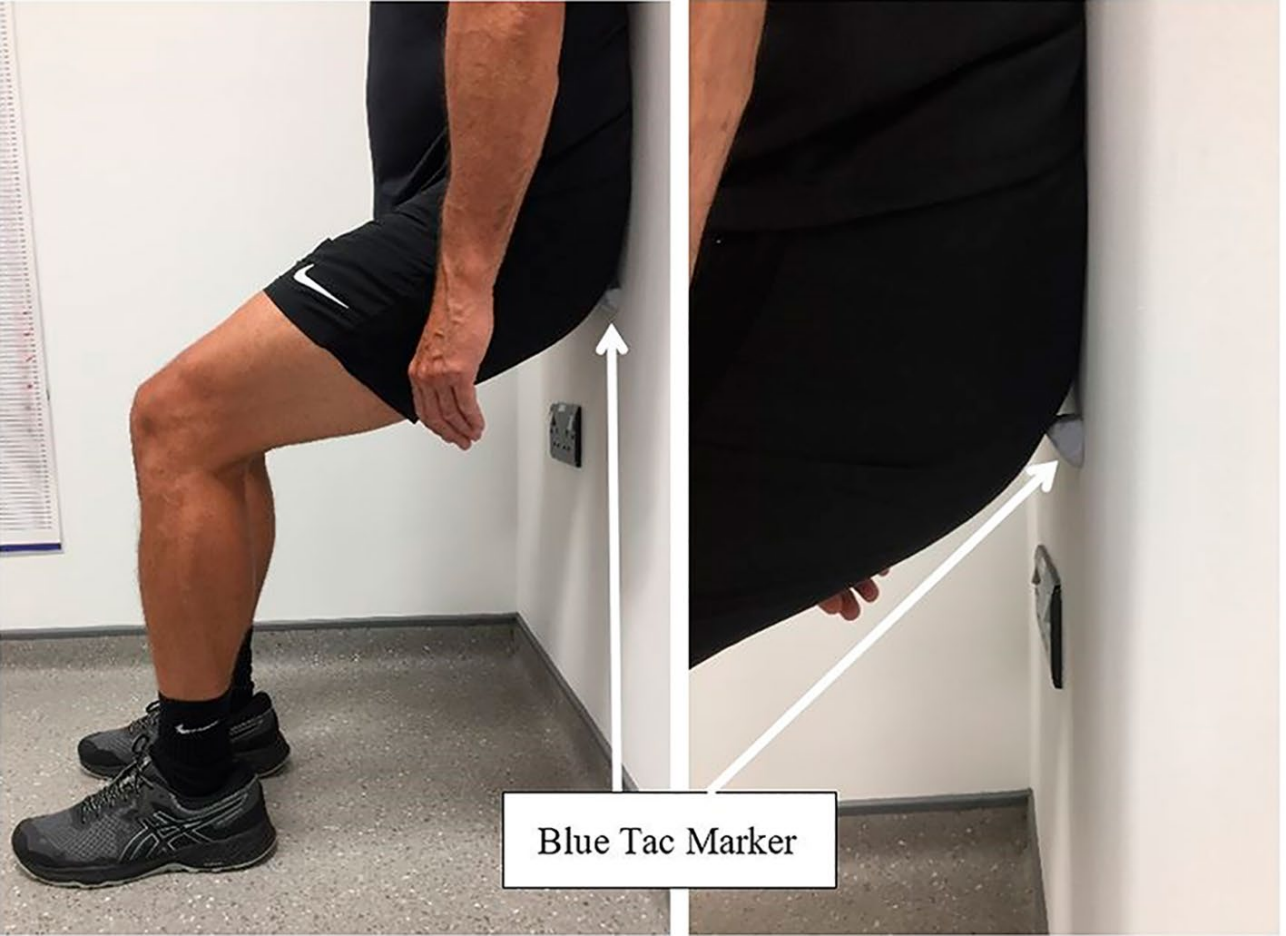
Squat height marker for RPE-EX group

During the baseline testing session, following the resting HR and BP measurements, RPE-EX participants conducted an RPE-based exercise intensity selection protocol. Based on pilot testing, the participants were firstly given 30-s to perform a wall squat, varying the squat height, to select a position that they estimated would elicit an RPE score of 4 after a 2-min contraction. Participants were instructed to change their foot position as they adjusted the squat height to keep their lower leg vertical. Once the participant had selected a squat height, they marked the position using some Blue Tac, stuck to mark the lowest point of contact with the wall (Fig. 2). The squat height, to the top of this marker, was then recorded for use in the home. Following two minutes rest, the participant completed four 2-min IWS contractions separated by 2-min rest periods. The first bout was conducted at the height selected during the 30-s period. At the end of each bout, participants gave an RPE score. This score was compared to a target RPE value and zone for each bout, developed based on pilot data and regression data from Lea et al. (2021a). Target values (and zones) were as follows; bout 1: RPE 4 (3.5–4.5), bout 2: RPE 5.5 (5–6), bout 3: RPE 7 (6.5–7.5), and bout 4: RPE 8.5 (8–9). If their RPE score fell outside of the target zone for a bout, the participant was instructed to change the squat height. Participants were encouraged to change the height 1 or 2 cm at a time and were reminded to change their foot position to maintain a vertical lower leg. Based on the final squat height and RPE score, after bout 4, participants were given a starting squat height for training session 1. Nine out of the 10 RPE-EX participants achieved an RPE score, within the target zone for bout 4, with 1 participant giving a final score of 7/10. This participant was instructed to further lower the squat height for the first training session.

#### IWS training sessions

The RPE-EX condition consisted of a 4-week home-based IWS training programme. Training was completed 3 days per week for 4 consecutive weeks (12 sessions in total) with 48-h between training sessions. Each training session was composed of 4 × 2-min bouts of IWS exercise separated by 2-min rest periods. Participants lowered their back down the wall whilst moving their feet forward until they reached the squat height marker, set to the height calculated at the end of the baseline testing session. To avoid the Valsalva manoeuvre, participants were instructed to breathe normally.

**Fig. 3:**
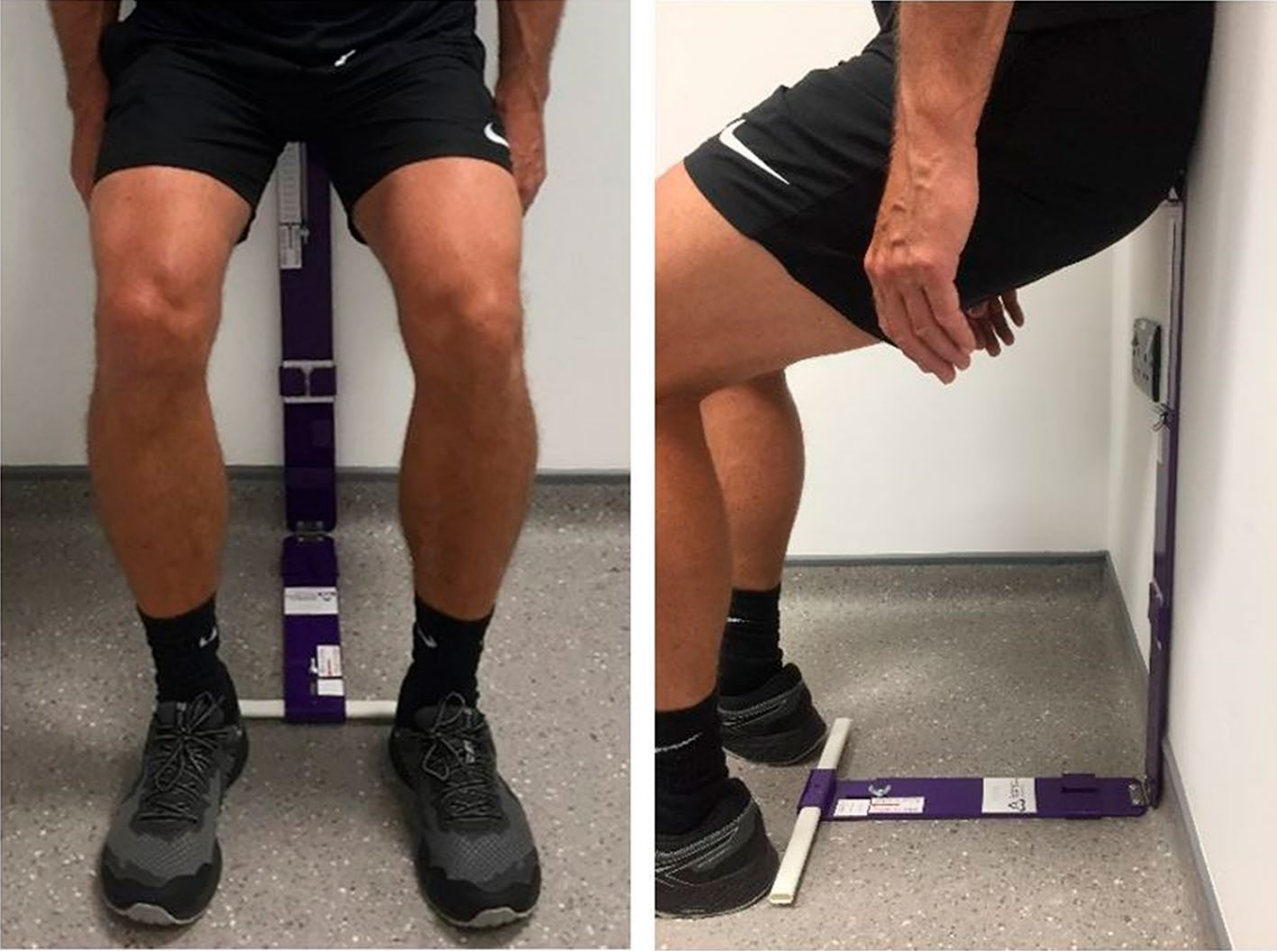
Isometric Exercise Scale modified with the target zone for peak RPE during training sessions

The first and last training sessions (sessions 1 and 12) were conducted in the laboratory, with no intervention from the researchers, whilst the other 10 training sessions were completed in the home. During each training session, participants recorded their squat height and RPE (Fig. 3) immediately following of each bout. Participants were instructed to use the same squat height for the duration of each training session; if the peak RPE result, at the end of bout 4, was outside the target zone then the starting squat height for the next session should be adjusted (± 1 or 2 cm at a time) accordingly. Based upon pilot work, it was suggested that after 1 or 2 sessions they should be able to pick a squat height that would remain constant for the rest of the 4-week intervention.

### HR-EX group

#### Baseline testing and training intensity prescription

Following resting HR and BP measurements, HR-EX participants completed an incremental isometric wall squat test (IIWST) to determine their isometric training intensity, as described in Wiles et al. (2017, 2018). The test required participants to lower their back down a sturdy wall and make small adjustments to their feet position until the required knee joint angle was reached, whilst maintaining a vertical lower limb and an erect trunk. The first stage began at 135° of knee flexion, and participants were instructed to hold this position for 2-min. Once each stage was complete, the knee joint angle was decreased by 10°. The angle was decreased every 2-min until the participant reached the end of the 95° stage or could no longer maintain the knee joint angle within 5° of the target value (volitional exhaustion). Following completion of the IIWST, knee joint angle was plotted against the mean HR data for the last 30-s of each stage. The equation for the relationship between HR and knee joint angle was used to predict the knee angle required to elicit 95% of HR peak (Devereux et al. 2010; Wiles et al. 2018). HR peak was defined as the mean HR for the last 30-s of the test. Once the training knee angle had been calculated, the squat height (lowest point of contact with the wall to floor) and foot distance (heel to wall) recorded at each stage were used to calculate the required measurements to replicate the required knee joint angle in the home.

#### IWS training sessions

**Fig. 4:**
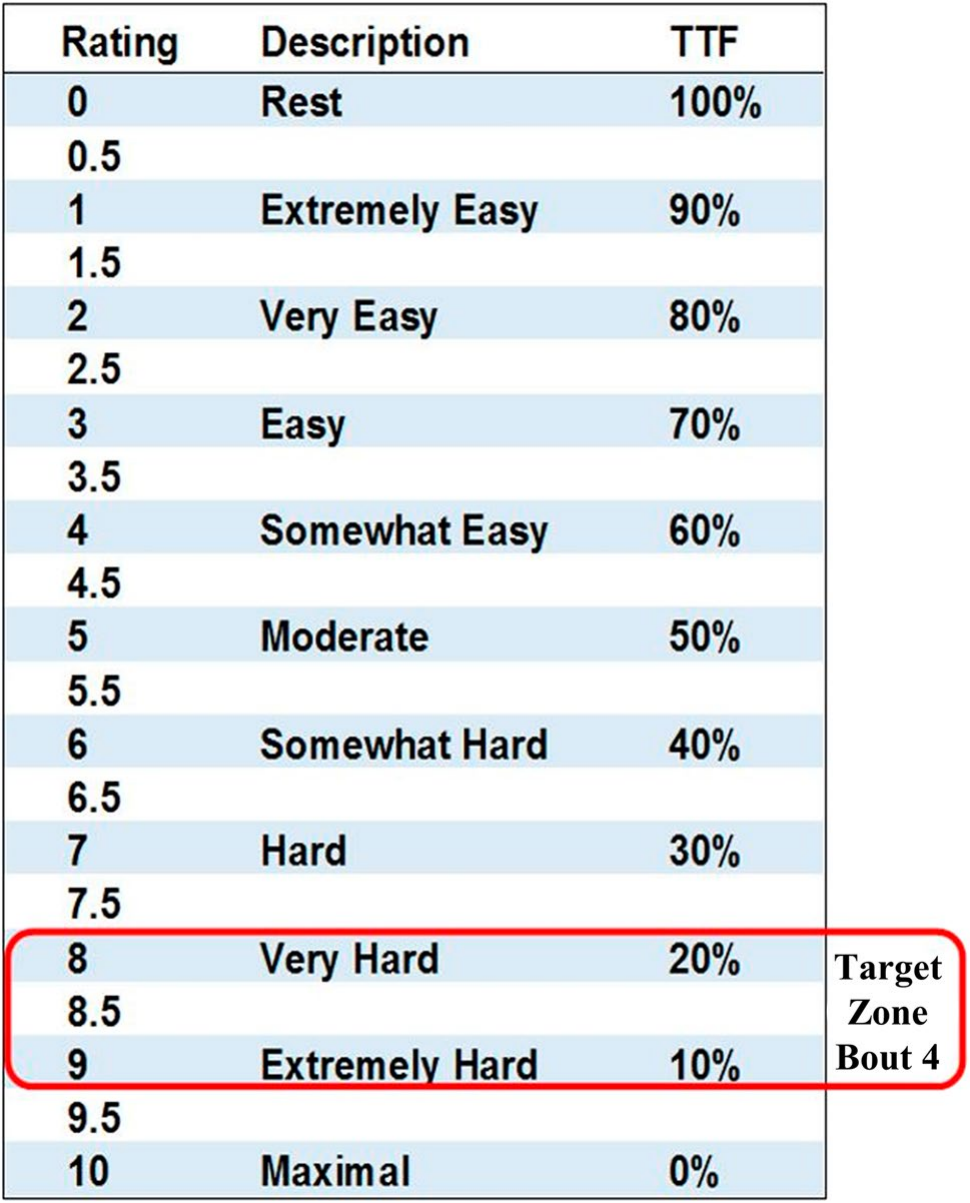
The Bend and Squat device in use during isometric wall squat exercise to set knee joint angle

The HR-EX condition consisted of a 4-week home-based IWS training programme, as described in Wiles et al. (2017). Training was completed 3 days per week for 4 consecutive weeks (12 sessions in total) with 48-h between training sessions. Each training session was composed of 4 × 2-min bouts of IWS exercise separated by 2-min rest periods. Participants were given a Bend and Squat device (made in-house) to control squat height and foot position to elicit the target knee joint angle (Fig. 4). The first and last training sessions (sessions 1 and 12) were conducted in the laboratory, with no intervention from the researchers, whilst the other 10 training sessions were completed in the home. During each training session, participants recorded their peak HR using a HR monitor (Polar RS400, Polar Electro Oy, Finland) and RPE (using the IES) immediately following of each bout. To avoid the Valsalva manoeuvre, participants were instructed to breathe normally. All training sessions were completed at the same participant-specific knee joint angle prescribed from the initial IIWST.

### Post intervention testing

At the end of training session 12, RPE-EX and HR-EX participants were given an AMBP cuff and were instructed to start a 24-h AMBP measurement between 24 and 48-h after completion of that session. Participants were then required to attend the laboratory for follow up measurements within 48–96 h from completion of the final training session. During this session, resting seated and supine HR and BP measurements were collected, as previously described.

### Data analysis

All data were analysed using the statistical package for social sciences (SPSS 24 release version for Windows, Armonk, NY). Before analysis, all data were checked for conformity with the parametric assumptions (Field 2009). Participant data (age, stature, mass and BMI) were assessed for differences between groups (3 levels: CON, HR-EX, RPE-EX) and time-points (2 Levels: baseline and post-intervention) using a 2-way analysis of variance (ANOVA). Post-hoc testing was completed using independent measures *t*-tests. Cardiovascular variables (resting and ambulatory) were assessed for differences between groups (3 levels: CON, HR-EX, RPE-EX) and time-points (2 levels: baseline and post training period) using 2-way ANOVA with post hoc paired samples or independent *t*-tests. Additionally, the mean differences (Delta score) in baseline and post values (post-result minus baseline result) were assessed between groups (3 levels) using one-way ANOVA with post-hoc independent *t*-tests (Seated BP, Ambulatory day and night BP) or independent samples Kruskal–Wallis tests with post-hoc Mann–Whitney *U* tests (supine BP and 24-h ambulatory BP), normal distribution dependant. Further to this, the clinical significance of any differences in BP (SBP and DBP) was determined by calculating the number of participants that achieved a BP reduction equal or greater than the minimal clinically important difference (MCID). According to the National Institute for Health and Care Excellence (NICE 2011), the MCID for SBP and DBP reduction is 5 mmHg, which is associated with a 10% reduction in the risk of mortality, cerebrovascular accident and myocardial infarction. Post hoc analyses were only conducted if ANOVA were significant using a Bonferroni adjustment for multiple comparisons, to reduce the risk of a type 1 error. An alpha level of < 0.05 was set as the threshold for statistical significance. All data are expressed as mean ± S.D, except for all supine BP measurements and 24-h ambulatory DBP, which are presented as median (interquartile range).

## Results

### Participant data

Participant anthropometric data for each study group are presented in Table 1. Participants in the RPE-EX group were significantly (*P* = 0.005) younger (− 14 years) than the participants in the HR-EX group, but not the CON group (*P* > 0.05). There were no other statistically significant differences between groups or time-points for any other variable (*P* > 0.05).

**Table 1:** Participant data pre- and post-intervention for each of the study groups

Variable	Control		HR-EX		RPE-EX	
	Pre	Post	Pre	Post	Pre	Post
Age (Years)	28 ± 4	28 ± 4	39 ± 15	39 ± 15	25 ± 4*	25 ± 4*
Stature (m)	178 ± 7	178 ± 7	175 ± 10	175 ± 10	180 ± 8*	180 ± 8*
Mass (kg)	82 ± 12	83 ± 12	85 ± 14	84 ± 15	77 ± 15*	77 ± 15*
BMI	26 ± 3	26 ± 3	28 ± 3	27 ± 3	24 ± 5*	24 ± 5*
Exercise/Week (Hours)	2 ± 2	2 ± 2	2 ± 2	2 ± 2	4 ± 3*	3 ± 3*

*Significantly lower than HR-EX at the same time-point. *BMI* Body Mass Index. Exercise/Week, number of hours of exercise in the previous week excluding any prescribed IWS exercise

### Resting measures

#### Seated measurements

**Fig. 5:**
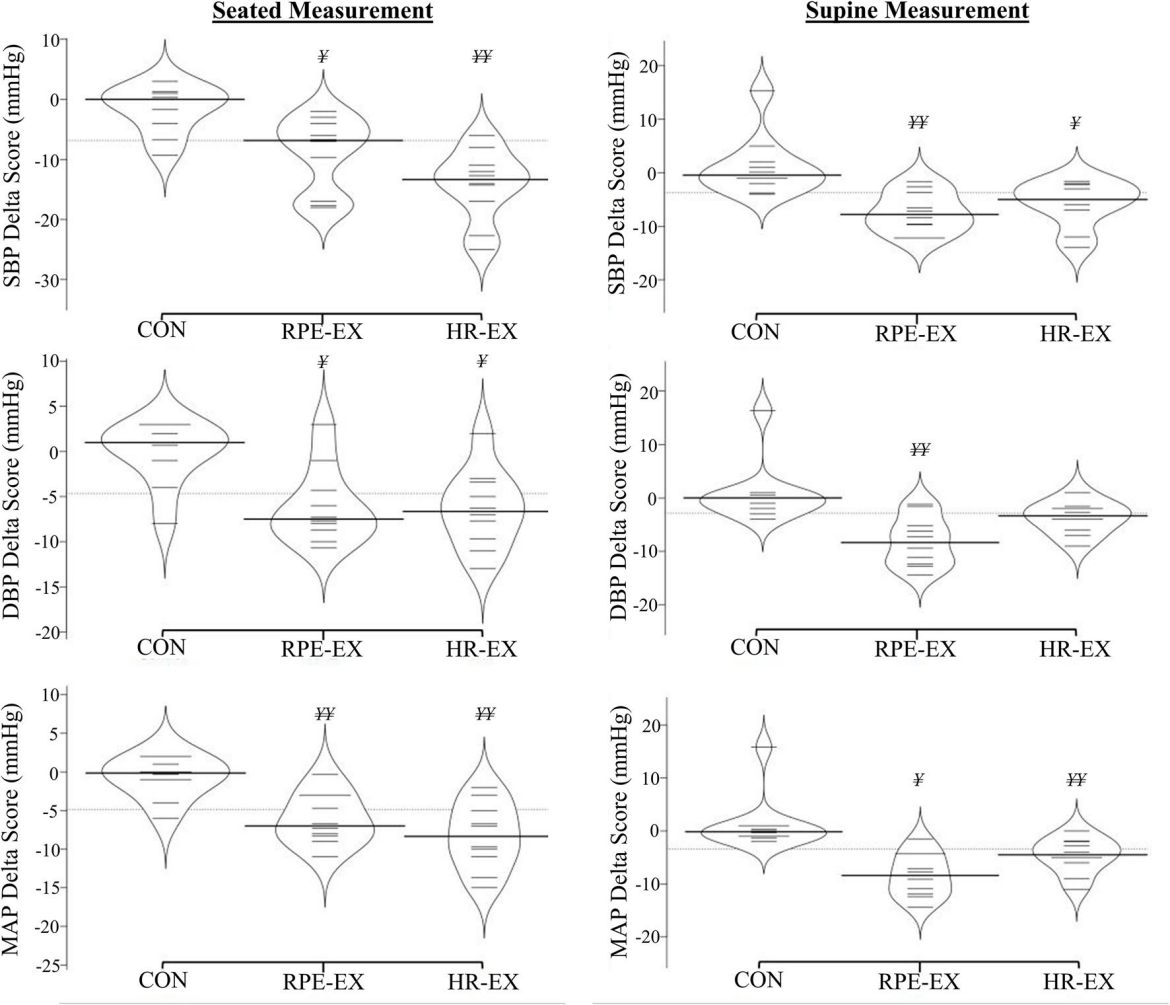
Group mean differences in seated and supine measurements of BP and HR following the IWS intervention (post result—baseline result). ^¥^*P* < 0.05, ^¥¥^*P* < 0.001 significantly greater reduction compared to the Control group

Resting clinic SBP, DBP and MAP results were significantly reduced (*P* < 0.001) in both intervention groups, following the 4-week intervention when compared to baseline (Table 2). The group mean reductions in each variable for the RPE-EX (SBP: − 9 ± 6, DBP: − 6 ± 4, MAP: − 6 ± 3 mmHg) and HR-EX (SBP: − 14 ± 6, DBP: − 6 ± 4, MAP: − 8 ± 4 mmHg) groups were significantly greater (*P* ≤ 0.001) than any changes in the CON group (Fig. 5). There were no significant differences between intervention groups in the magnitude of the BP reductions (*P* > 0.05). However, at baseline HR-EX had significantly higher SBP (*P* < 0.001) and MAP (*P* = 0.001) results compared to RPE-EX (Table 2), but after the interventions, there were no significant differences between groups (*P* > 0.05).

**Table 2:** Group mean resting BP and HR results pre and post 4-week intervention

Variable	CON		HR-EX		RPE-EX	
	Pre	Post	Pre	Post	Pre	Post
Seated resting measurements						
SBP (mmHg)	128.6 ± 8.5	127.0 ± 11.0	134.0 ± 4.0^††^	119.8 ± 5.8**	125.1 ± 5.3^††^	116.0 ± 3.8**
DBP (mmHg)	80.6 ± 7.0	80.4 ± 8.0	82.6 ± 5.3	76.2 ± 5.7**	77.3 ± 8.4	71.2 ± 7.6**
MAP (mmHg)	98.2 ± 8.3	97.4 ± 9.1	101.2 ± 3.0^†^	92.9 ± 4.3**	94.4 ± 4.4^†^	88.3 ± 4.1**
HR (b.min^−1^)	64.3 ± 7.1	64.6 ± 9.1	62.5 ± 7.3	62.1 ± 10.5	71.5 ± 16.7	67.0 ± 11.3
Supine resting measurements						
SBP (mmHg)	113.9 ± 11.8	115.1 ± 10.7	124.9 ± 11.2	119.1 ± 10.7*	115.3 ± 5.6	107.9 ± 5.3**
DBP (mmHg)	69.2 ± 13.3	70.0 ± 12.0	77.9 ± 5.8^†^	74.2 ± 6.4*^†^	70.1 ± 3.7^†^	62.0 ± 5.3**^†^
MAP (mmHg)	85.8 ± 11.0	87.1 ± 9.7	94.4 ± 6.8	89.7 ± 7.3*	87.8 ± 2.9	79.4 ± 4.8**
HR (b.min^−1^)	57.8 ± 4.9	57.8 ± 4.7	57.9 ± 7.0	57.6 ± 9.0	58.8 ± 9.6	57.4 ± 9.5

*P < 0.05

**P < 0.001 significant with-in group differences

^†^*P* < 0.05

^††^*P* < 0.001 significant difference compared to the other intervention group at the same time-point

#### Supine measurements

Supine resting SBP, DBP and MAP results were significantly lower, in the RPE-EX (*P* < 0.001) and HR-EX (*P* = 0.002) groups, following the intervention when compared to baseline (Table 2). The group mean reductions in each BP variable (Fig. 5) for RPE-EX (SBP: − 8 (− 5), DBP: − 8 (− 7), MAP: − 8 (− 7) mmHg) were significantly greater than the changes seen in the CON group (*P* < 0.001). Likewise, the group mean reductions in SBP (− 5 (− 4) mmHg) and MAP (− 5 (− 4) mmHg) seen in the HR-EX group, were significantly greater than any changes present in the CON group, whereas the group mean reduction in DBP for the HR-EX group (− 3 (− 4) mmHg; Fig. 5) was not statistically significant when compared to the CON (*P* > 0.05). There were no significant differences between the RPE-EX and HR-EX groups in the group mean reductions in any BP variables (*P* > 0.05).

### Ambulatory measures

**Fig. 6:**
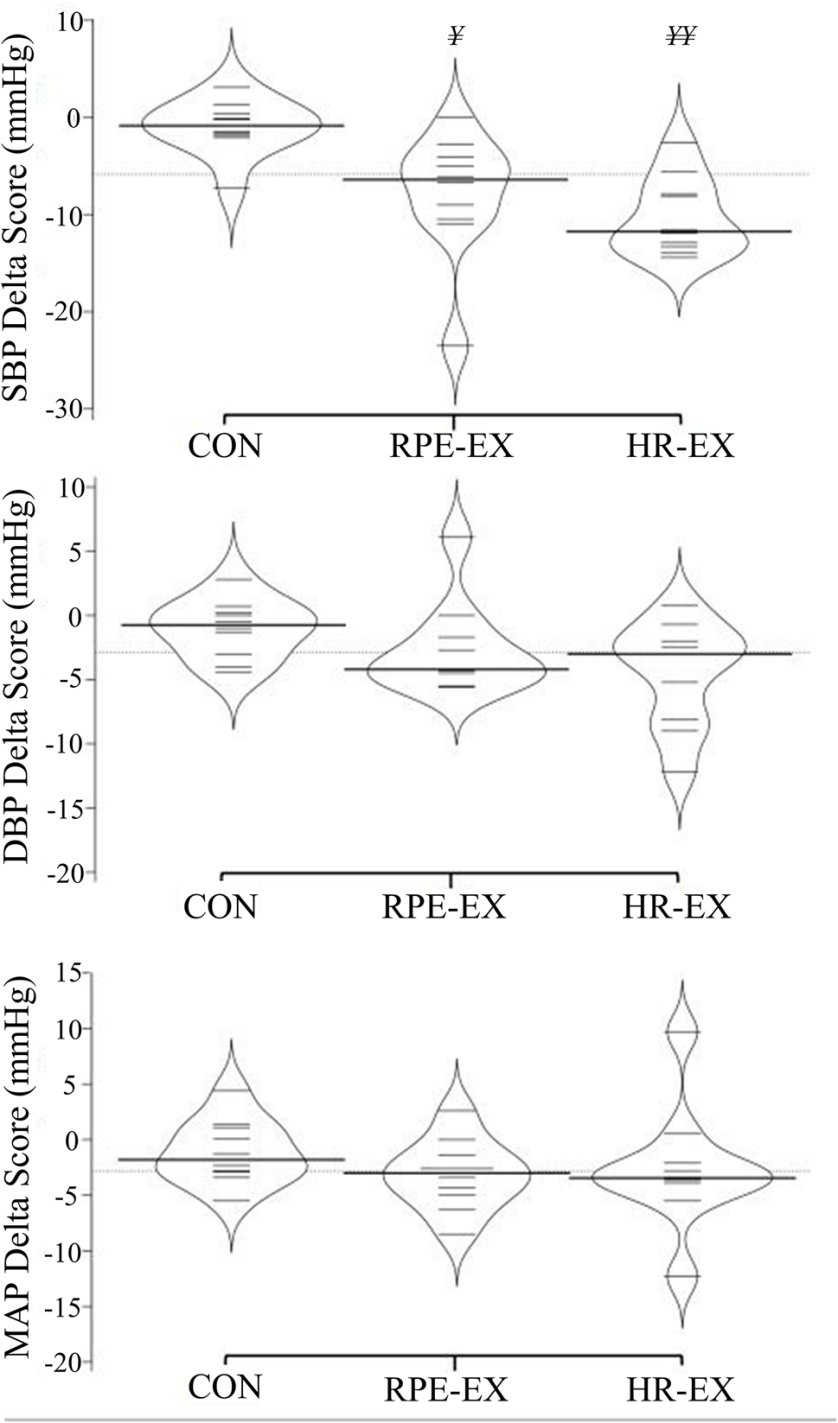
Group mean differences in 24-h ambulatory BP and HR measurements following the IWS intervention (post result—baseline result). ^¥^*P* < 0.05, ^¥¥^*P* < 0.001 significantly greater reduction compared to the Control group

Both intervention groups showed significantly lower 24-h (RPE-EX: *P* = 0.004; HR-EX: *P* < 0.001) (Fig. 6), daytime (RPE-EX: *P* = 0.007; HR-EX: *P* < 0.001), and night-time (RPE-EX: *P* = 0.012; HR-EX: *P* = 0.004) ambulatory SBP results post-intervention when compared to the baseline (Table 3). The RPE-EX group did not show any differences in pre-post DBP measurements (*P* > 0.05) but did show a significantly lower post result for 24-h MAP readings (*P* = 0.012). Conversely, the HR-EX group showed significantly lower post-intervention ambulatory DBP results for the 24-h (*P* = 0.007) and night-time (*P* = 0.001) measurements, but not for the daytime measurements (*P* > 0.05). Additionally, the HR-EX group did not show any significant differences in pre-post MAP results (*P* > 0.05). Finally, the HR-EX group showed significantly lower post-intervention results for 24-h HR (*P* = 0.006), whilst no differences in HR were seen in any other group or measurement time (Table 3). There were no significant between group differences for any variable or time-point (*P* > 0.05).

**Table 3:** Pre- and post-intervention group mean 24-h, day, and night ambulatory BP and HR

Variable	Control		HR-EX		RPE-EX	
	Pre	Post	Pre	Post	Pre	Post
24 Hour Ambulatory BP						
SBP (mmHg)	126.6 ± 5.6	125.6 ± 6.7	130.8 ± 5.6	120.5 ± 7.3**	134.6 ± 17.2	126.7 ± 17.7*
DBP (mmHg)	69.7 ± 5.0	68.6 ± 5.1	73.0 ± 7.5	68.5 ± 5.4*	73.3 ± 8.0	70.7 ± 7.5
MAP (mmHg)	87.3 ± 5.8	86.2 ± 4.9	89.0 ± 5.4	86.3 ± 4.1	92.5 ± 9.7	89.3 ± 9.6*
HR (b.min^−1^)	70.9 ± 8.2	70.4 ± 10.0	70.7 ± 7.3	65.2 ± 7.8*	73.5 ± 10.3	73.1 ± 10.7
Day Ambulatory BP (08:00–22:00)						
SBP (mmHg)	128.2 ± 4.5	127.6 ± 6.3	132.9 ± 6.8	124.1 ± 7.3**	139.2 ± 21.3	130.3 ± 19.7*
DBP (mmHg)	73.0 ± 4.9	71.6 ± 5.2	75.3 ± 7.4	72.1 ± 3.9	78.3 ± 10.7	75.5 ± 9.2
MAP (mmHg)	90.9 ± 5.4	89.3 ± 4.9	93.0 ± 7.4	89.7 ± 4.3	98.3 ± 13.3	93.9 ± 11.4
HR (b.min^−1^)	72.2 ± 6.9	73.0 ± 11.5	74.4 ± 7.2	70.4 ± 9.4	77.1 ± 11.3	75.5 ± 10.7
Night Ambulatory BP (00:00–06:00)						
SBP (mmHg)	113.4 ± 10.5	112.1 ± 6.9	112.5 ± 9.7	105.8 ± 10.6*	118.9 ± 15.0	111.2 ± 14.1*
DBP (mmHg)	59.6 ± 5.5	60.2 ± 5.5	59.7 ± 2.4	56.2 ± 4.6*	60.4 ± 6.9	57.4 ± 6.6
MAP (mmHg)	76.3 ± 6.3	77.0 ± 5.3	76.6 ± 3.8	73.4 ± 5.9	78.8 ± 6.7	74.8 ± 6.7
HR (b.min^−1^)	62.1 ± 6.9	62.8 ± 6.4	65.1 ± 7.4	61.4 ± 7.3	62.0 ± 12.7	65.2 ± 13.6

*P < 0.05

**P < 0.001 significant with-in group differences

### Minimum clinically important difference

The number of participants showing a MCID (− 5 mmHg) or greater in SBP or DBP measurements was calculated for each BP measurement type. In the CON group, 1 participant showed a difference greater or equal to the MCID in seated SBP and DBP, and ambulatory SBP, but did not show a MCID in any supine measurement. In both intervention groups, 100% of the participants showed an MCID or greater in one or more of the BP measurements taken. In addition to this, 8 of the RPE-EX participants were pre-hypertensive at baseline based upon the seated clinic BP measurements; following the intervention, only 2 participants remained pre-hypertensive. Likewise, all 10 HR-EX participants were classified as pre-hypertensive at baseline, with only 3 participants remaining pre-hypertensive following the intervention.

## Discussion

The key findings of this pilot study were the clinically significant reductions in one or more BP measure in 100% of the intervention participants; this included significant reductions in both seated and supine resting measurements and significant reductions in ambulatory SBP. Blood pressure reductions matching or exceeding the MCID are associated with significant reductions in the risk of developing hypertension and cardiovascular disease (Prospective Studies Collaboration 2002; Whelton et al. 2002), as well as reduced risk of myocardial infarction, stroke and mortality (NICE 2019). In addition, six RPE-EX and seven HR-EX participants would be reclassified as normotensive following completion of the 4-week IWS interventions. This reclassification to normotensive is associated with further risk reduction, in addition to receiving the MCID reductions alone (Vasan et al. 2001). This demonstrates the prophylactic importance of this IWS intervention, as shown in the current study, and its ability to lower participants back into a normotensive classification.

The current study demonstrated statistically significant reductions in resting supine (SBP: − 8 (− 5), DBP: − 8 (− 7), MAP: − 8 (− 7) mmHg) and seated (SBP: − 9 ± 6, DBP: − 6 ± 4, MAP: − 6 ± 3 mmHg) BP in the RPE-EX group; with similarly significant reductions in the supine (SBP: − 5 (− 4), MAP: − 5 (− 4) mmHg) and seated (SBP: − 14 ± 6, DBP: − 6 ± 4, MAP: − 8 ± 4 mmHg) BP measurements in the HR-EX group. When comparing the results of the current study to previous research, 4 weeks of IWS exercise produced resting BP reductions comparable or greater than those previously shown following 8 weeks of isometric hand grip (Badrov et al. 2013; Millar et al. 2013; Carlson et al. 2016) and isometric leg extension (Devereux et al. 2010; Baross et al. 2013). Moreover, these reductions were greater than the averages previously reported (SBP: − 5 mmHg and DBP: − 4 mmHg) in a meta-analysis of laboratory-based IE interventions (Inder et al. 2016), and in the most recent meta-analysis (SBP-6/7, DBP-3, MA-4/5 mmHg), that included a mixture of hypertensive (medicated and un-medicated), pre-hypertensive and normotensives participants (Smart et al. 2019). This may demonstrate a benefit of IWS exercise, possibly due to the additional isometric recruitment of postural and stabilising muscles when holding the constant position wall squat (Contreras 2013), which are not required when conducting isometric leg extension or isometric hand grip exercise which isolates the quadriceps and forearm respectively. Interestingly, isometric hand grip but not isometric leg exercise has now been endorsed by the American Heart Association as an adjuvant BP lowering treatment (McGowen et al. 2017). Furthermore, the resting BP reductions shown in the current study were comparable to the reductions demonstrated following aerobic exercise interventions, SBP: − 11 mmHg and DBP: − 5 mmHg (Börjesson et al. 2016), a mode of exercise that is already widely recommended for the reduction of resting BP (Pescatello et al. 2004). Thus, this further demonstrates the potential usefulness and importance of this new RPE prescribed intervention, which can elicit BP reductions comparable to interventions that are already endorsed, whilst being more accessible and shorter in duration.

The 24-h AMBP results showed significant reductions in SBP (RPE-EX − 8 ± 7 mmHg; HR-EX – 10 ± 4 mmHg) and non-statistically significant reductions in DBP (RPE-EX − 4 (− 3) mmHg; HR-EX − 3 (− 5) mmHg) and MAP (RPE-EX − 3 ± 3 mmHg; HR-EX − 3 ± 6 mmHg). Previously, Somani et al. (2017) showed smaller reductions in 24-h SBP, of ∼4 mmHg following isometric hand grip training and showed no differences in DBP or MAP results. However, Somani et al. used young normotensive adults, which may have meant they had limited capacity for AMBP change, when compared to the predominately pre-hypertensive cohort recruited in the current study. Indeed, when studying a pre-hypertensive population, Taylor et al. (2019) showed similar reductions in 24-h SBP (− 11.8 ± 3.5 mmHg) and demonstrated significant reductions in DBP (− 5.6 ± 3.3 mmHg) and MAP (− 5.7 ± 4.4 mmHg). Whilst the current study was unable to show statistically significant reductions in DBP and MAP in a mixed normotensive and pre-hypertensive cohort, clinically significant reductions in 24-h SBP, as shown in both intervention groups, represents a significant reduction in the risk of all-cause mortality (NICE 2011), with a greater association to health outcomes than resting clinical measurements (Benegas et al. 2018).

Comparison of each IWS protocol suggests that they were largely equally effective; indeed, there were no significant differences in any of the reductions between intervention groups. Ostensibly, the mean seated SBP reductions were greater, although non-statistically significantly, in the HR-EX group compared to RPE-EX (− 14 ± 6 mmHg vs. − 9 ± 6 mmHg respectively); however, this was likely due to the significantly greater baseline SBP recorded in HR-EX group. Alternatively, it is possible that the HR-EX method allows tighter control of and therefore a more consistent training stimulus between participants when compared to the RPE-EX method. Arguably, this is to be expected, given the use of maximal testing, HR monitoring and the lack of subjectivity in the exercise intensity measurement with the HR-EX method. However, it should be noted that 100% of RPE-EX participants did show MCID reductions. Therefore, more research is required to examine whether there is any advantage to the HR-EX method. In addition, even if the BP reductions and cardiovascular improvements using the RPE-EX method are more modest, it may still be that the accessibility of the RPE-EX method to a larger proportion of the target population, will make it an overall more effective intervention. It is also possible that participants that are introduced to this type of intervention via the more accessible RPE-EX method will then move on to using the more clinical HR-EX method for further improvements. Indeed, the aim of the RPE intervention is not necessarily to supersede the previous intervention, thus rendering it obsolete, but rather is to increase the overall impact of IWS interventions, by working concurrently to increase overall participation.

A limitation to this study was the significantly greater baseline BP results and age in the HR-EX group when compared to the RPE-EX participants. It was not possible to counterbalance all participant characteristics in this study as too many existed for the number of participants; therefore, participants were randomised into groups prior to the first testing session (and before baseline BP measurement), with only sex being counterbalanced. Differences in baseline BP must be considered when analysing the effect of training interventions, as it has previously been demonstrated that more pronounced BP reductions occur in participants with higher baseline BP (Millar et al. 2007). Likewise, increased age may have affected the results as it is common for SBP to increase progressively with age (Franklin et al. 1997), often due to atherosclerosis within the arteries, decreased luminal diameter and reduced arterial elasticity (Mancia et al. 2013). As such, participants over the age of 45 years have been shown to present larger reductions in MAP than those under 45 years, whilst no differences were shown in SBP or DBP reductions (Inder et al. 2016). Taking this into account, it seems likely that the two interventions in this study were equally effective, although further research with larger sample sizes is required to examine any possible differences.

## Conclusion

Significant reductions in resting and ambulatory BP were seen following a 4-week home-based IWS intervention, using RPE to prescribe and monitor exercise intensity, with clinically important BP reductions in 100% of participants. As shown in previous studies, the HR prescribed IWS intervention, also elicited significant and clinically important BP reductions. These results confirm the effectiveness of the previous IWS methodology and demonstrates the potential of this new, more accessible, RPE-based method, as an effective lifestyle intervention for the prevention of hypertension in both normotensive and pre-hypertensive participants.

### Supplementary Information

Below is the link to the electronic supplementary material.

Supplementary file1 (DOCX 16 kb)

## Abbreviations

ANOVA: Analysis of variance
AMBP: Ambulatory blood pressure
BP: Blood pressure
CON: Control group
DBP: Diastolic blood pressure
EMG: Electromyography
HR: Heart rate
HR-EX: Exercise intervention group with intensity controlled using heart rate
IE: Isometric exercise
IES: Isometric exercise scale
IWS: Isometric wall squat
MAP: Mean arterial pressure
MCID: Minimum clinically important difference
RPE: Ratings of perceived exertion
RPE-EX: Exercise intervention group with intensity controlled using ratings of perceived exertion
SBP: Systolic blood pressure
