# Supplementary material for: The implementation of a home-based isometric wall squat intervention using ratings of perceived exertion to select and control exercise intensity: a pilot study in normotensive and pre-hypertensive adults
Source: Eur J Appl Physiol. 2023 Jul 17;124(1):281–93. doi: 10.1007/s00421-023-05269-2 (PMC10786991; doi:10.1007/s00421-023-05269-2)
Supplement: Supplementary file 1 — Supplementary file1 (DOCX 16 kb) [file 421_2023_5269_MOESM1_ESM.docx]

# **The implementation of a home-based isometric wall squat intervention using ratings of perceived exertion to select and control exercise intensity: a pilot study in normotensive and pre-hypertensive adults.**

John W. D. Lea^1^, Jamie M. O’Driscoll^1^ & Jonathan D. Wiles^1^*

*1-School of Human and Life Sciences, Canterbury Christ Church University, Kent, UK
*Corresponding Author – Email Address: jim.wiles@canterbury.ac.uk*

**Supplementary Table 1:** Significant 2-way ANOVA p-values.

| **Variables** | **P-Value** | **Table** | **Figure** |
| --- | --- | --- | --- |
| Participant Age | 0.004 | 1 | - |
| Seated Resting SBP | 0.000 | 2 | 5 |
| Seated Resting DBP | 0.002 | 2 | 5 |
| Seated Resting MAP | 0.000 | 2 | 5 |
| Supine Resting SBP | 0.001 | 2 | 5 |
| Supine Resting DBP | 0.001 | 2 | 5 |
| Supine Resting MAP | 0.000 | 2 | 5 |
| 24-hour Ambulatory SBP | 0.000 | 3 | 6 |
| 24-hour Ambulatory DBP | 0.000 | 3 | 6 |
| 24-hour Ambulatory MAP | 0.004 | 3 | 6 |
| 24-hour Ambulatory HR | 0.028 | 3 | - |
| Day Ambulatory SBP | 0.005 | 3 | - |
| Night Ambulatory SBP | 0.019 | 3 | - |
| Night Ambulatory DBP | 0.047 | 3 | - |
